# Lifetime Trauma History and Cognitive Functioning in Major Depression and Their Role for Cognitive-Behavioral Therapy Outcome

**DOI:** 10.32872/cpe.4105

**Published:** 2021-09-30

**Authors:** Lena Schindler, Tobias Stalder, Clemens Kirschbaum, Franziska Plessow, Sabine Schönfeld, Jürgen Hoyer, Sebastian Trautmann, Kerstin Weidner, Susann Steudte-Schmiedgen

**Affiliations:** 1 Faculty of Psychology, Technische Universität Dresden, Dresden, Germany; 2 Department Erziehungswissenschaften und Psychologie, Universität Siegen, Siegen, Germany; 3 Neuroendocrine Unit, Department of Medicine, Massachusetts General Hospital and Harvard Medical School, Boston, MA, USA; 4 Department of Psychology, Lund University, Lund, Sweden; 5 Institute of Clinical Psychology and Psychotherapy, Technische Universität Dresden, Dresden, Germany; 6 Department of Psychology, Medical School Hamburg, Hamburg, Germany; 7 Department of Psychotherapy and Psychosomatic Medicine, Medical Faculty Carl Gustav Carus, Technische Universität Dresden, Dresden, Germany; Philipps-University of Marburg, Marburg, Germany

**Keywords:** major depression, lifetime trauma history, working memory, interference susceptibility, conflict adaptation, autobiographical memory, cognitive-behavioral therapy

## Abstract

**Background:**

While cognitive-behavioral therapy (CBT) is the gold-standard psychological treatment for major depression (MD), non-response and lacking stability of treatment gains are persistent issues. Potential factors influencing treatment outcome might be lifetime trauma history and possibly associated primarily prefrontal-cortex- and hippocampus-dependent cognitive alterations.

**Method:**

We investigated MD and healthy control participants with (MD+T+, n = 37; MD-T+, n = 39) and without lifetime trauma history (MD+T-, n = 26; MD-T-, n = 45) regarding working memory, interference susceptibility, conflict adaptation, and autobiographical memory specificity. Further, MD+T+ (n = 21) and MD+T- groups (n = 16) were re-examined after 25 CBT sessions, with MD-T- individuals (n = 34) invited in parallel in order to explore the stability of cognitive alterations and the predictive value of lifetime trauma history, cognitive functioning, and their interaction for treatment outcome.

**Results:**

On a cross-sectional level, MD+T+ showed the highest conflict adaptation, but MD+T- the lowest autobiographical memory specificity, while no group differences emerged for working memory and interference susceptibility. Clinical improvement did not differ between groups and cognitive functioning remained stable over CBT. Further, only a singular predictive association of forward digit span, but no other facets of baseline cognitive functioning, lifetime trauma history, or their interaction with treatment outcome emerged.

**Discussion:**

These results indicate differential roles of lifetime trauma history and psychopathology for cognitive functioning in MD, and add to the emerging literature on considering cognitive, next to clinical remission as a relevant treatment outcome.

## Highlights

- Conflict adaptation was highest in MD with lifetime trauma history.
- Autobiographical memory specificity was lowest in MD without lifetime trauma history.
- No differential treatment response was found in MD with and without lifetime trauma history.
- There were no changes of cognitive functioning over CBT, irrespective of lifetime trauma history.
- Only singular predictive value of cognitive functioning for CBT success emerged.

Meta-analyses suggest cognitive-behavioral therapy (CBT) as the gold-standard psychological treatment for major depression (MD; e.g., Barth et al., 2013; Cuijpers et al., 2014), a condition characterized by depressed mood and loss of motivation together with behavioral alterations such as reduced activity and disturbed sleep (Diagnostic and Statistical Manual of Disorders – Fifth Edition; American Psychiatric Association, 2013). However, a substantial patient subgroup fails to achieve clinically significant symptom improvement, with non-response and dropout rates of approximately 34% and 25%, respectively (for meta-analytic data, see Cuijpers et al., 2014; Hans & Hiller, 2013). This highlights the need to enhance our understanding of factors associated with psychopathology and treatment outcome, allowing an optimization of CBT effects and reduction of dropout rates. Here, trauma history is frequently discussed, defined as exposure to actual or threatened death, serious injury, or sexual violence (American Psychiatric Association, 2013). Particularly for childhood trauma in MD, associations with poorer therapy response, longer remission time, and greater need for additional medication are relatively well-researched (for review and meta-analytic data, see Nanni et al., 2012; Nemeroff, 2016; Teicher & Samson, 2013). Notably, lifetime trauma, including childhood, adulthood, or both types of trauma, has been far less well studied, except for one study suggesting negative associations of both childhood and adulthood adversity with therapy outcome in MD (Miniati et al., 2010).

Importantly, lifetime trauma history is assumed to co-occur with neurobiological (e.g., Kolassa & Elbert, 2007; Sherin & Nemeroff, 2011) and cognitive alterations (e.g., Vasterling & Arditte Hall, 2018). However, data on this and its influence on therapy success in the context of MD and trauma is sparse. In particular, primarily prefrontal-cortex- and hippocampus-dependent functioning have received attention (McIntyre et al., 2013; Rock et al., 2014; Snyder, 2013; Snyder & Hankin, 2019). Regarding the former, of importance might be working memory (WM) as a facet of executive functioning (EF) relevant for temporal maintenance (usually assessed by the repetition of a list of numbers) and manipulation (usually assessed by the repetition of a list of numbers in a backward fashion) of content necessary for current tasks (Diamond, 2013). Accumulating evidence suggests impaired WM in patients with MD (for reviews, see Snyder, 2013; Snyder & Hankin, 2019). Further, one study reported childhood trauma to predict performance in a compound WM score of information maintenance and manipulation in both patients with MD and healthy controls (Saleh et al., 2017), but another found no WM differences with respect to information maintenance or manipulation in patients with MD with or without childhood trauma (Dannehl et al., 2017).

An EF domain considered to be even more impaired in MD (e.g., Snyder, 2013; Snyder & Hankin, 2019) is the ability to suppress irrelevant and/or interfering response tendencies while pursuing mentally represented goals (i.e., inhibitory control, Diamond, 2013). Typically, this is studied via the well-known *Simon task* (Simon, 1990), where the inhibition of a response following a task-irrelevant visual stimulus is necessary as a different response is required. The resulting additional performance costs (i.e., slower reaction times [RTs] and/or increased percentages of error [PEs]) compared to trials with matching automatic and required tendencies comprise the so-called Simon effect as a measure of interference susceptibility (Simon, 1990). After response conflicts, inhibitory control is typically increased, leading to a decreased impact of task-irrelevant information compared to trials not following conflicts. The resulting difference in the Simon effect is termed conflict adaptation (Botvinick et al., 2001). In MD, particularly this conflict adaptation according to task demands is suggested to be increased (van Steenbergen et al., 2012). Notably, previous work from our group revealed similar findings for patients with posttraumatic stress disorder (PTSD) and, albeit less clearly, trauma-exposed controls (Schindler et al., 2020; Steudte-Schmiedgen et al., 2014), encouraging research on the interaction of trauma and MD.

Of note, there is an abundance of studies suggesting not only EF, but also mainly hippocampally-driven overgeneral memory retrieval (OGM) to be a central correlate of MD (for meta-analytic data, see, e.g., Sumner et al., 2010). This increased recall of overgeneral (e.g., “I am happy when meeting friends”) instead of specific autobiographical memories (e.g., “I was happy on July 8 when I met friends”; Williams et al., 2007) is also prevalent in PTSD, with trauma history a potential shared mechanism (Moore & Zoellner, 2007; Ono et al., 2016; Sumner et al., 2010; Williams et al., 2007). However, previous contrasting of trauma-exposed and non-exposed individuals with MD (notably, again only focusing on childhood trauma) provided mixed results, with one study finding OGM only in trauma-exposed (Aglan et al., 2010) and another only in non-exposed individuals (Kuyken et al., 2006).

Next to these cross-sectional findings of certain alterations of EF and autobiographical memory domains, and the possible mediating role of trauma history in MD, it is plausible to assume that such alterations show significant change over psychotherapy. However, the vast majority of studies could not detect any changes of the cognitive alterations described above over psychotherapy/combined psycho- and pharmacotherapy (for WM, see, e.g., Beblo et al., 1999; Lahr et al., 2007; for inhibitory control, see, e.g., Schmid & Hammar, 2013; but Ajilchi et al., 2016; for OGM, see, e.g., Peeters et al., 2002). Thus, a current meta-analysis (Bernhardt et al., 2019) rather support the suggestions from previous reviews (e.g., Bernhardt et al., 2019; Köhler et al., 2015; Moore & Zoellner, 2007; Snyder & Hankin, 2019) of high stability of such alterations even after clinical remission, with improvements not exceeding task-specific practice effects. While previous data on cognitive markers as predictors for clinical outcome in the context of pharmacotherapy is promising (Groves et al., 2018), research on CBT is outstanding, except for initial studies suggesting a predictive value of enhanced autobiographical memory specificity (Sumner et al., 2010), but not interference susceptibility (Goodkind et al., 2016). However, while lifetime trauma history is assumed to be associated with both therapy outcome (e.g., Nemeroff, 2016; Teicher & Samson, 2013) and cognitive alterations (e.g., Vasterling & Arditte Hall, 2018) in MD, a combined investigation is still pending.

Hence, the aim of the current study was to examine (i) lifetime trauma history and (ii) facets of cognitive functioning (i.e., WM, interference susceptibility, conflict adaptation, and OGM) as well as (iii) their interaction in the context of MD symptomatology and therapy success. Due to the inconclusive literature on the interplay of lifetime trauma history and MD for cognitive functioning, our first step was to study respective baseline alterations in MD and healthy control participants with (MD+T+, *n* = 37; MD-T+, *n* = 39)^1^1MD+T+ = patients with MD with lifetime trauma history; MD+T- = patients with MD without lifetime trauma history; MD-T+ = patients without MD with lifetime trauma history; MD-T- = patients without MD and without lifetime trauma history. and without lifetime trauma history (MD+T-, *n* = 26; MD-T-, *n* = 45). Specifically, we aimed to (1) investigate whether the previously found effect of lifetime trauma history on conflict adaptation (Schindler et al., 2020; Steudte-Schmiedgen et al., 2014) is also visible in MD and (2) shed light on the conflicting evidence regarding OGM (Aglan et al., 2010; Kuyken et al., 2006). Further, we assessed clinical and cognitive treatment outcome under consideration of lifetime trauma history by re-examining patients with MD with (MD+T+, *n* = 21) and without lifetime trauma history (MD+T-, *n* = 16) after 25 CBT sessions. In order to account for practice effects, non-traumatized healthy control individuals (MD-T-, *n* = 34) were re-invited in parallel. Here, we hypothesized (3) poorer treatment outcome for MD+T+ than for MD+T- individuals. Based on recent meta-analytic evidence (Bernhardt et al., 2019), we aimed to examine whether we could confirm the finding of (4) no changes of cognitive functioning over CBT, irrespective of lifetime trauma history, also for the tasks studied here. On a last note, we aimed to (5) exploratorily study the predictive value of cognitive functioning for CBT outcome.

## Method

### Participants and Procedures

Recruitment was conducted within the outpatient unit of the Institute of Clinical Psychology and Psychotherapy of the Technische Universität Dresden, as well as via flyers and local advertisements. Individuals were included in the study if they were aged between 18 and 65 years, not pregnant (women), and did not report any severe physical diseases (e.g., cancer, encephalopathy) over the past five years. Further exclusion criteria concerned hair-related and endocrine factors due to biomarker analyses reported elsewhere (e.g., glucocorticoid medication; Steudte et al., 2013; Steudte-Schmiedgen et al., 2014). The presence of MD and any other DSM-IV (American Psychiatric Association, 2007) mental disorders was assessed using the standardized Munich Composite International Diagnostic Interview (*DIA-X/M-CIDI;* Wittchen & Pfister, 1997) conducted by therapists of the outpatient unit or trained research team members and confirmed by an experienced clinical psychologist. Twenty-eight participants from the MD groups showed psychiatric comorbidities within the last 12 months (one: *n* = 15, two: *n* = 8, three or more: *n* = 5). Those encompassed specific (*n* = 12) or social phobia (*n* = 13), somatoform disorders (*n* = 6), panic disorder with or without agoraphobia (*n* = 8), generalized anxiety (*n* = 3), obsessive-compulsive (*n* = 2), adjustment (*n* = 2), or eating disorders (*n* = 1).

An assignment to the MD groups was based on a current primary 12-month MD diagnosis and no 12-month diagnosis of substance abuse or dependence (except for nicotine) or any lifetime diagnoses of psychosis, severe depressive disorder with psychotic symptoms, or bipolar disorder. Notably, individuals meeting the lifetime diagnostic criteria for PTSD were also excluded from the study, in order to allow insights into the role of lifetime trauma exposure *per se* for cognitive functioning in MD. Participants were included in the control group if they did not report any lifetime mental disorders according to the *DIA-X/M-CIDI* stem questions and the *Mini International Neuropsychiatric Interview* (*M.I.N.I.;* Sheehan et al., 1998). Participants were further classified as exposed or non-exposed to lifetime trauma based on the *Posttraumatic Stress Diagnostic Scale* (*PDS;* Ehlers, Steil, Winter, & Foa, 1996). For an allocation to the T+ groups, both the “objective” A1 (“actual or threatened death or serious injury or a threat to the physical integrity of oneself or others”) and the “subjective” A2 criterion (“intense fear, helplessness or horror”) had to be met, following the DSM-IV requirements that qualify life events as traumatic (American Psychiatric Association, 2007). The control groups are the same as in the parallel study on patients with PTSD (Schindler et al., 2020). For further participant characteristics, see Table 1 and Supplementary Materials (type of lifetime trauma history).

**Table 1:** Baseline Demographic, Health-Related, and Clinical Characteristics of Patients With Major Depression With (MD+T+) and Without (MD+T-) as well as Controls With (MD-T+) and Without (MD-T-) Lifetime Trauma History

Participants’ characteristics	MD+T+ (*n* = 37)	MD+T- (*n* = 26)	MD-T+ (*n* = 39)	MD-T- (*n* = 45)	Test statistic	*p*
Demographics						
Age (*M*, *SD*)	37.59 (11.91)	39.27 (11.8)	41.46 (12.82)	35.31 (13.8)	*F*(3, 143) = 1.71	.167
Female sex (%)	27 (73)	17 (65.4)	32 (82.1)	38 (84.4)	${Χ}_{3}^{2}$ = 4.34	.227
Highest educational status					${Χ}_{\mathrm{12}}^{2}$ = 22.26	.035
Academic degree (%)	7 (18.9)	2 (8)^a^	16 (41)	11 (24.4)		
Professional training/college degree (%)	10 (27)	12 (48)^a^	13 (33.3)	15 (33.3)		
A level (%)	13 (35.1)	5 (20)^a^	7 (17.9)	16 (35.6)		
High school diploma/lower (%)	7 (18.9)	6 (24)^a^	3 (7.7)	3 (6.7)		
Smoking (%)	8 (21.6)	11 (42.3)	4 (10.3)	10 (22.2)	${Χ}_{3}^{2}$ = 9.24	.026
Physical disease (%)	19 (51.4)	11 (42.3)	17 (43.6)	12 (26.7)	${Χ}_{3}^{2}$ = 5.58	.134
Regular medication (%)	21 (56.8)	16 (61.5)	11 (28.2)	8 (17.8)	${Χ}_{3}^{2}$ = 21.02	< .001
Psychiatric (%)^b^	16 (43.2)	12 (46.2)	0	0		
Non-psychiatric (%)	10 (27)	8 (30.8)	11 (28.2)	8 (17.8)		
*PDS* score *(M, SD)*	12.4 (11.45)	n.a.	4.56 (6.18)	n.a.	*t*_54.72_ = 3.68	.001
*THQ* number of A1 traumatic events (*M, SD*)	4.3 (2.5)	2.81 (2.08)	4.21 (3.17)	1.33 (1.21)	*F*(3, 143) = 14.87	< .001^I^
*THQ* frequency of A1 traumatic events (*M, SD*)	7.33 (6.71)^c^	6.12 (5.1)	7.26 (8.42)	2.64 (3.79)	*F*(3, 143) = 5.25	.002^II^
*CTQ* score *(M, SD)*	39.59 (10.48)	37.45 (10.83)	37.94 (13)	29.02 (4.87)	*F*(3, 143) = 9.43	< .001^III^
*BDI-II* score *(M, SD)*	20.78 (9.11)	22.15 (8.75)	5.1 (6.8)	4.52 (0.67)	*F*(3, 143) = 66.66	< .001^IV^

*Note.* PDS = Posttraumatic Stress Diagnostic Scale; THQ = Trauma History Questionnaire; CTQ = Childhood Trauma Questionnaire; BDI-II = Beck Depression Inventory-II.

^a^refers to *n* = 25. ^b^included antidepressants (*n* = 27), anticonvulsives (*n* = 3), neuroleptics (*n* = 2), sedatives (*n* = 2). ^c^refers to *n* = 36.

^I^MD+T+ = MD-T+ > MD-T- (*p*s < .001), with MD+T- in between. ^II^MD+T+ = MD-T+ > MD-T- (*p*s ≤ .006), with MD+T- in between. ^III^MD+T+ = MD-T+ = MD-T+ > MD-T- (*p*s ≤ .005). ^IV^MD+T+ = MD+T->MD-T+ = MD-T- (*p*s ≤ .001).

CBT for MD groups was conducted within the outpatient unit based on established manuals (Hautzinger, 1998, 2008) and supervised by experienced therapists. After 25 sessions, MD+T+ and MD+T- patients were re-invited for clinical and cognitive testing, with MD-T- participants being contacted in a parallel fashion (no difference regarding months between assessments: *M* = 13.5, *SD* = 3.86; *M* = 11.56, *SD* = 4.03; and *M* = 14.76, *SD* = 6.97, respectively; *F*(2, 68) = 1.78, *p* = .177, $\eta_{p}^{2}$ = .05). Among the 63 patients with MD examined at baseline, 6 (9.5%) were only interested in the cross-sectional study, 16 (25.4%) dropped out of CBT, and 41 (65.1%) completed therapy. Between those who dropped out of CBT and those who did not, no differences emerged regarding pre-treatment clinical variables (all *p*s ≥ .219). All participants had provided written informed consent before study inclusion. The study protocol was approved by the ethics committee of the Technische Universität Dresden (EK 65022010) and conducted in accordance with the Declaration of Helsinki.

### Clinical and Psychological Measures

Self-developed questionnaires were applied for socio-demographic (age, sex, education status) and health-related variables (smoking, chronic physical diseases, regular medication intake). Depressive symptoms over the previous two weeks were assessed via the *Beck Depression Inventory-II* (*BDI-II*, Hautzinger et al., 2006). The *PDS* (Ehlers et al., 1996) provided insights into the presence or absence of lifetime trauma history and the severity of symptoms associated with posttraumatic stress according to *DSM-IV* criteria. The *Trauma History Questionnaire* (*THQ,* Maercker, 2002) provided an overview over number and frequency of potentially traumatic events fulfilling the *DSM-IV* A1, but not A2 criterion (Hooper et al., 2011). Furthermore, to obtain information on the severity of childhood maltreatment (irrespective of fulfilling *DSM-IV* A criteria), the *Childhood Trauma Questionnaire* (*CTQ,* Gast et al., 2001) was used. At follow-up, patients with MD additionally received the revised version of the *Questionnaire of Changes in Experience and Behavior* (*Veränderungsfragebogen des Erlebens und Verhaltens VEV-R;* Zielke & Kopf-Mehnert, 2001). This allowed a classification of patient-evaluated therapy effects via 42 items of opposite polarity (e.g., “Compared with the time prior to initiation of therapy, I feel more relaxed/no change/more tense.”) into three categories (i.e., symptom improvement, no change, and worsening).

### Cognitive Tasks

WM was examined using the *Wechsler Memory Scale* digit span task (Wechsler, 1997). Participants repeated a series of numbers read out loud by the experimenter in a forward (information maintenance) or backward fashion (information manipulation). Interference susceptibility and conflict adaptation were assessed by a number version of the *Simon task* (Fischer et al., 2008). In brief, participants categorized the numbers 1 to 9, except 5, as smaller or larger than five by pressing a left (Alt) or right (Alt Gr) key on a QWERTZ keyboard with their left or right index finger, respectively. Although task-irrelevant, stimulus location automatically facilitates the pressing of the corresponding response button, either in accordance, or in conflict with the required action, resulting in compatible and incompatible trials, respectively. The resulting difference in RTs and PEs comprises the Simon, and the typical reduction of interference susceptibility after conflict trials the conflict adaptation effect (Botvinick et al., 2004; Simon, 1990). Participants completed a 16-trial practice, followed by three 64-trial test blocks, resulting in 192 test trials (for further details, see Schindler et al., 2020; Steudte-Schmiedgen et al., 2014). Indices for interference susceptibility (I – C) and conflict adaptation [(cI – cC) – (iI – iC)] (lowercase letters: compatibility of the previous, uppercase letters: compatibility of the current trial, larger values indicating more pronounced effects) were calculated (van Steenbergen et al., 2010).

Autobiographical memory specificity was assessed via the standardized *Autobiographical Memory Test* (Williams & Broadbent, 1986). Participants were instructed to read words out loud (practice phase: three neutral words, testing phase: five positive and five negative words in a pseudo-randomized order, starting with a positive word and alternating valence) and briefly describe a related specific autobiographical memory. The words were randomly chosen from a word pool from a previous study (Schönfeld & Ehlers, 2006) matched for word frequency, emotionality, imagery, and pleasantness (apart from positive words rated as more pleasant than negative ones; Hager & Hasselhorn, 1994), with different sets used at baseline and follow-up. Answers were tape-recorded, transcribed and coded by trained research assistants. As an outcome variable, the number of specific memories was used, defined as having happened at a particular place and time more than one week ago and having lasted for one day or less. If no answer was provided within 30 seconds, the trial was considered an omission. For assessing inter-rater-reliability, a second, independent rater re-assessed a random sample (10%) of the tape-recorded sequences, resulting in κ = .76 for the baseline and κ = .82 for the follow-up assessment.

### Statistical Analyses

Analyses were conducted via SPSS for Windows, version 25 (IBM, Armonk, NY), R (R Core Team, 2017), and STATA 15.1 (StataCorp LLC, 2017). Cross-sectional group comparisons were carried out via univariate analyses of variance (ANOVAs; continuous variables) and Χ^2^ contingency tables (dichotomous variables). For the Simon task, the first trial of each block (1.6%), posterror trials (3%), target repetitions (11.3%), and, for RT analyses, error trials (3%) were excluded. AMT data from one MD+T- and one MD-T- participant were missing.

For longitudinal analyses, as a first step, participants from the MD+T+, MD+T-, and MD-T- groups with available longitudinal data were re-examined regarding baseline demographic and clinical differences. Simon task data from one MD+T+ and three MD-T- participants, and AMT data from one MD+T- and two MD-T- participants were missing. Again, the first trial of each block (1.6%), posterror trials (baseline: 2.8%, follow-up: 2.7%), target repetitions (baseline: 11.5%, follow-up: 10.9%) and, for RT analyses, error trials (baseline: 2.8%, follow-up: 2.7%) were excluded. Repeated-measures ANOVAs with time [2, baseline vs. follow-up] as within-subject and group [3, MD+T+ vs. MD+T- vs. MD-T-] as between-subject factor were applied to assess clinical and cognitive changes over CBT.

Exploratory linear/logistic regression analyses were conducted for examining the predictive value of lifetime trauma history (*PDS*; yes/no) for changes of depressive symptom severity (*BDI-II*) and dropout from care as core outcome measures, respectively. Due to the small sample size for the longitudinal analyses, and the high correlations between depressiveness (*BDI-II*) and the subjectively evaluated therapy effects (*VEV-R*, *r* = -.66, *p* < .001) at the follow-up assessment, we decided to omit the *VEV-R* from the predictive analyses. For the *BDI-II*, a change score was computed by subtracting baseline from follow-up values, and baseline values were included as a covariate to the regression analyses. As a second step, baseline cognitive performance (centered around the mean to avoid multicollinearity issues), and, as a third, the interaction of lifetime trauma history (yes/no) and baseline cognitive performance were added to the model.

Whenever hypothesis testing referred to one major cognitive domain (i.e., EF and learning/memory) and were not exploratory in nature, Holm-Bonferroni correction (Holm, 1979) for family-wise error (FWER) per respective domain was applied. As the assumptions of conventional GLMs (ANOVA, linear regression) are frequently violated in psychological data possibly leading to poor power and inaccurate effect sizes (Field & Wilcox, 2017), we repeated hypothesis testing using robust regressions. These drop GLM assumptions by using a robust sandwich estimation of standard errors, down-weighting observations with large residuals, and omitting outlying residuals (Royall, 1986). Predictive analyses were repeated using mixed-effects regressions with random intercept parameter addressing regression to the mean, which can otherwise yield biased results (Oberg & Mahoney, 2007). However, due to the higher prevalence and familiarity of conventional GLMs in the field, whenever both analyses yielded the same results, conventional GLMs were reported.

## Results

### Sample Characteristics, Clinical Symptomatology, and Baseline Cognitive Functioning

The groups were well-matched regarding age, sex, and physical diseases (all *p*s ≥ .134, see Table 1). However, group differences emerged for educational status (${Χ}_{\mathrm{12}}^{2}$ = 22.26, *p* = .035) and smoking (${Χ}_{3}^{2}$ = 9.24, *p* = .026). Furthermore, both clinical groups reported higher medication intake than the non-clinical ones (${Χ}_{3}^{2}$ = 21.02, *p* < .001), mainly driven by psychiatric medication. However, including these variables as covariates did not change the cross-sectional results. For depressive symptom severity (*BDI-II*), both MD+T- and MD+T+ individuals reported higher levels than the control groups (all *p*s ≤ .001), with post-hoc analyses indicating no difference between them. For number and frequency of *DSM-IV* A1 traumatic events, both MD+T+ and MD-T+ scored higher than MD-T- individuals, with MD+T- individuals in between (*THQ*, all *p*s ≤ .006). For the severity of childhood maltreatment, both MD+ groups as well as the MD-T+ participants scored higher than the MD-T- group (*CTQ*, all *p*s ≤ .005).

**Figure 1:**
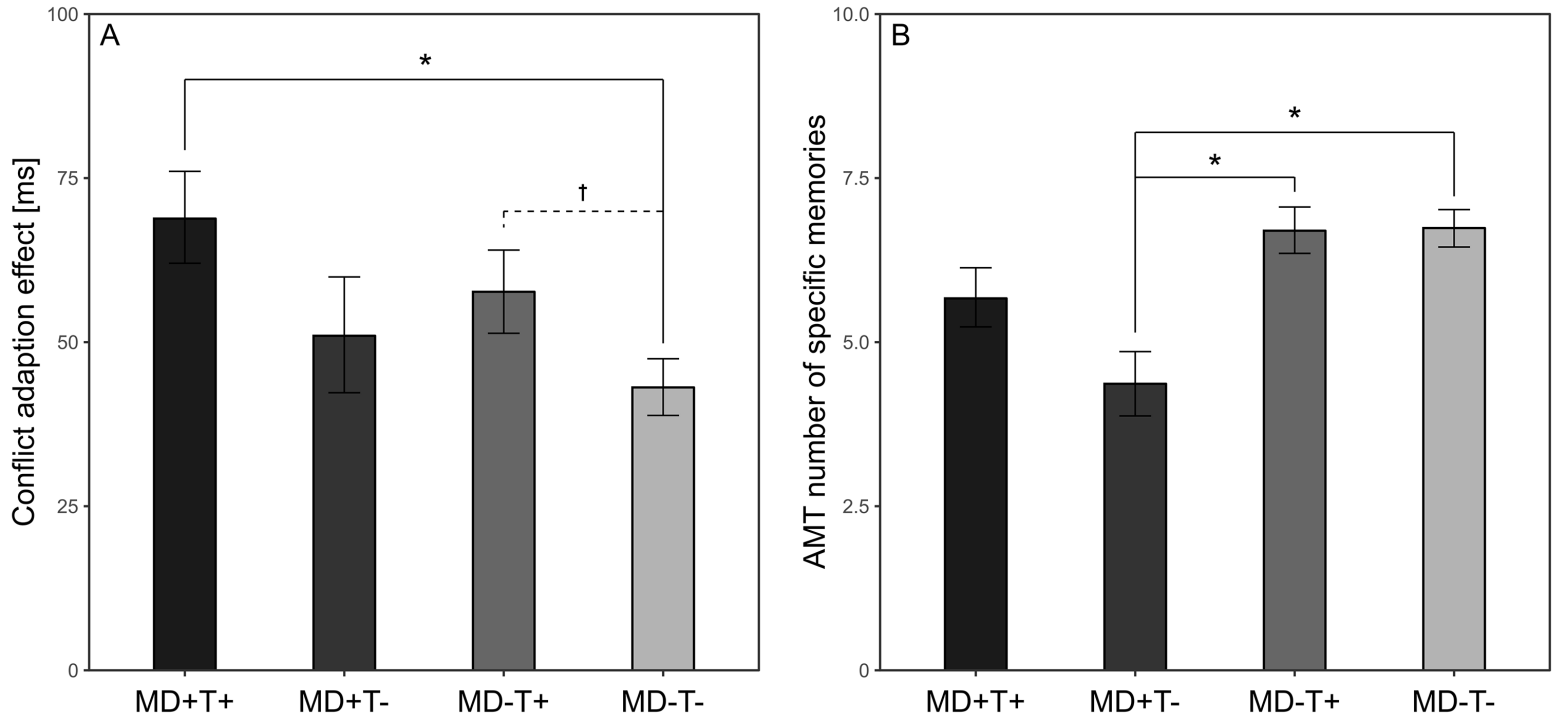
Mean (± *SEM*) (A) Conflict Adaptation of Median RTs (Simon Task) and (B) Specificity of Autobiographical Memory (Autobiographical Memory Test) of Patients With Major Depression With (MD+T+) and Without (MD+T-) as well as Controls With (MD-T+) and Without (MD-T-) Lifetime Trauma History at Baseline *Note.* *p < .05, †*p* < .10, dotted lines indicate differentiating results between general linear and robust models.

No group differences emerged for forward, backward, and overall digit span (all *p*s ≥ .283, see Table 2). For the Simon task, groups differed regarding conflict adaptation of median RTs with a medium effect size, *F*(3, 143) = 3.23, *p* = .024, $\eta_{p}^{2}$ = .063, 90% CI [0, .12], see Figure 1), with higher levels in MD+T+ compared to MD-T- individuals (*p* = .017) and no other differences (all *p*s ≥ .43). Neither for conflict adaptation of mean PEs, nor for interference susceptibility did group differences emerge (all *p*s ≥ .424). Regarding OGM, for positive and negative words and the overall score, MD+T- participants scored lower than both MD-T+ and MD-T- ones with, again, medium effect sizes (all *p*s ≤ .002), and no other differences (all *p*s ≥ .118).

While OGM results remained stable after Holm-Bonferroni correction for FWER, the group difference for conflict adaptation of median RTs lost statistical significance (*p* = .168). Applying robust regressions did not considerably change the results, except for the difference between MD-T+ and MD-T- participants regarding conflict adaptation of median RTs and the interference effect of median RTs emerging as non-significant trends, β = -15.2, 95% CI [-30.7, 0.2], *p* = .053 and β = -11.5, 95% CI [-24.2, 1.2], *p* = .076).

**Table 2:** Baseline Working Memory in the Digit Span Task, Interference and Conflict Adaptation Effects of Means of Median Reaction Times (RTs) and Percentage of Errors (PE) in the Simon Task, and Autobiographical Memory Specificity in the Autobiographical Memory Test (AMT) of Patients With Major Depression With (MD+T+) and Without (MD+T-) as well as Controls With (MD-T+) and Without (MD-T-) Lifetime Trauma History

Facet of cognitive functioning	MD+T+ (*n* = 37)	MD+T- (*n* = 26)	MD-T+ (*n* = 39)	MD-T- (*n* = 45)	Test statistic	*p*	$\eta_{p}^{2}$ 90% CI [*LL*, *UL*]	Adjusted *p* (Holm-Bonferroni correction)
Digit Span								
Total	17.57 (3.52)	17.77 (3.82)	17.33 (3.74)	18.62 (3.03)	*F*(3, 143) = 1.1	.351	.023 [0, .06]	1
Forward	9.84 (1.94)	9.81 (1.98)	9.72 (1.75)	10.13 (1.7)	*F*(3, 143) = 0.41	.746	.009 [0, .03]	1
Backward	7.73 (2.06)	7.96 (2.36)	7.62 (2.4)	8.49 (2.14)	*F*(3, 143) = 1.28	.283	.026 [0, .07]	1
Simon task: RT								
Interference effect	27.32 (29.05)	24.88 (28.2)	31.87 (28.54)	21.99 (24.81)	*F*(3, 143) = 0.94	.424	.019 [0, .05]	1
Conflict adaptation effect	68.93 (42.42)	51.15 (44.22)	57.74 (39.49)	43.14 (28.83)	*F*(3, 143) = 3.23	.024^I^	.063 [0, .12]	.168
Simon task: PE								
Interference effect	1.83 (3.39)	1.73 (3.57)	2.05 (4.48)	1.32 (3.57)	*F*(3, 143) = 0.27	.846	.006 [0, .02]	1
Conflict adaptation effect	5.23 (5.88)	5 (5.79)	5.26 (7.12)	4.6 (6.42)	*F*(3, 143) = 0.1	.962	.002 [0, 0]	1
AMT number of specific memories								
Total	5.68 (2.72)	4.36 (2.46)^a^	6.69 (2.18)	6.73 (1.86)^b^	*F*(3, 141) = 7.16	< .001^II^	.132 [.05, .21]	< .001
Positive cues	2.92 (1.44)	2.4 (1.35)^a^	3.49 (1.36)	3.57 (1.23)^b^	*F*(3, 141) = 5.2	.002^III^	.1 [.02, .17]	.002
Negative cues	2.76 (1.54)	1.96 (1.34)^a^	3.21 (1.2)	3.16 (1.16)^b^	*F*(3, 141) = 5.72	.001^IV^	.11 [.03, .18]	.002

*Note.* Data are presented as *M* (*SD*). CI = confidence interval; *LL* = lower level; *UL* = upper level; RT = median reaction times; PE = percentages of error.

^a^refers to *n* = 25. ^b^refers to *n* = 44.

^I^MD+T+ > MD-T- (*p* = .017). ^II^MD+T- < MD-T+ = MD-T- (*p*s ≤ .001). ^III^MD+T- < MD-T+ = MD-T- (*p*s ≤ .011). ^IV^MD+T- < MD-T+ = MD-T- (*p*s ≤ .002).

### Clinical and Cognitive Treatment Outcome Under Consideration of Lifetime Trauma History

MD+T+ (*n* = 21), MD+T- (*n* = 16), and MD-T- participants (*n* = 34) available for longitudinal analyses did not differ regarding baseline demographic/health-related characteristics (all *p*s ≥ .136, see Supplementary Materials), except for higher medication intake in both MD groups (${Χ}_{2}^{2}$ = 13.9, *p* = .001). However, including it as a covariate did not affect the longitudinal results. MD+T+ individuals reported a higher number of *DSM-IV* A1 traumatic events (*THQ*) than MD+T- ones, which, in turn, reported more than MD-T- individuals (all *p*s ≤ .036). With respect to their frequency (*THQ*), as well as for childhood maltreatment severity (*CTQ*), both MD+ groups scored higher than the MD-T- one (all *p*s ≤ .035 and all *p*s ≤ .002, respectively).

Notably, while CBT led to substantial clinical improvements, MD+T+ and MD+T- individuals did not differ regarding depressive symptom changes (*BDI-II*), subjectively evaluated therapy effects (*VEV-R*), and percentage of dropouts (all *p*s ≥ .605, see Table 3). Furthermore, no cognitive improvements over CBT in the clinical groups emerged (all *p*s ≥ .272, see Table 3). However, for digit span, medium-to-large time effects indicated better performance at follow-up over all groups (all *p*s ≤ .009). Robust regressions yielded similar results.

**Table 3:** Clinical Improvement and Changes in Working Memory in the Digit Span Task, Interference and Conflict Adaptation Effects of Means of Median Reaction Times (RTs) and Percentage of Errors (PE) in the Simon Task, and Autobiographical Memory Specificity in the Autobiographical Memory Test (AMT) Between Baseline and Follow-Up Assessment in Patients With Major Depression With (MD+T+) and Without (MD+T-) as well as Controls Without (MD-T-) Lifetime Trauma History

Participants’ characteristics	MD+T+ (*n* = 21)		MD+T- (*n* = 16)		MD-T- (*n* = 34)		Test statistic	*p*	$\eta_{p}^{2}$ 90% CI [*LL*, *UL*]	Adjusted *p* (Holm-Bon-ferroni cor-rection)
	T1	T2	T1	T2	T1	T2				
*BDI-II* score *(M, SD)*	21.67 (7.72)	12.14 (9.52)	23.12 (8.83)	11.25 (8.19)	3.97 (4.76)	4.59 (5.3)	*F*_2, 68_ = 23.54^a,b^	< .001^I^	.409 [.25, .52]	-
*BDI-II* change score *(M, SD)*	-9.52 (8.48)		-11.88 (7.69)		0.62 (5.32)					-
*VEV-R* symptom improvement (%)	16 (76.2)		13 (86.7)		n.a.		${Χ}_{2}^{2}$ = 1.01	.605	n.a.	-
Dropouts (%)	9 (29)		7 (26.9)		n.a.		${Χ}_{2}^{2}$ = 0.31	1.00	n.a.	-
Digit Span										
Total	18.57 (3.16)	19.81 (4.09)	18.13 (3.57)	18.94 (3.09)	18.59 (3.2)	20.38 (3.54)	*F*_2, 68_ = 0.77^a^	.47	.022 [0, .09]	1
Forward	10.1 (1.73)	10.81 (2.21)	9.81 (1.8)	9.94 (1.39)	10 (1.67)	10.94 (1.98)	*F*_2, 68_ = 1.17^a^	.318	.033 [0, .11]	1
Backward	8.48 (1.94)	9 (2.15)	8.31 (2.3)	9 (2.03)	8.59 (2.29)	9.44 (2.21)	*F*_2, 68_ = 0.18^a^	.833	.005 [0, .04]	1
Simon task: RT										
Interference effect	26.48 (29.66)^b^	20.05 (26.93)^b^	20.38 (27.09)	15.78 (23.59)	20.71 (25.65)^c^	24.48 (24.12)^c^	*F*_2, 64_ = 1.33^a^	.272	.04 [0, .12]	1
Conflict adaptation effect	66.6 (29.95)^b^	60.1 (31.4)^b^	54.88 (50.85)	65.84 (35.65)	41.02 (23.4)^c^	50.32 (37.03)^c^	*F*_2, 64_ = 0.96^a^	.387	.029 [0, .1]	1
Simon task: PE										
Interference effect	2.24 (1.84)^b^	1.55 (3.27)^b^	1 (3.35)	1.45 (2.58)	1.31 (4.05)^c^	2.41 (2.76)^c^	*F*_2, 64_ = 1.23^a^	.299	.037 [0, .12]	1
Conflict adaptation effect	4.76 (5.49)^b^	4.22 (6.16)^b^	4.4 (5.75)	4.24 (5.12)	5 (6.26)^c^	3.32 (6.2)^c^	*F*_2, 64_ = 0.25^a^	.78	.008 [0, .05]	1
AMT number of specific memories										
Total	6.05 (2.71)	6.67 (2.31)	4.47 (2.13)^d^	4.53 (2.23)^d^	6.75 (2)^e^	6.25 (2.24)^e^	*F*_2, 65_ = 1.21^a^	.305	.036 [0, .11]	
Positive cues	3.1 (1.38)	3.29 (1.31)	2.47 (1.3)^d^	2.4 (1.3)^d^	3.63 (1.29)^e^	3.19 (1.42)^e^	*F*_2, 65_ = 1.2^a^	.308	.036 [0, .11]	
Negative cues	2.95 (1.47)	3.38 (1.32)	2 (1.13)^d^	2.13 (1.13)^d^	3.12 (1.24)^e^	3.06 (1.16)^e^	*F*_2, 65_ = 0.64^a^	.529	.019 [0, .08]	

*Note.* Data is presented as *M* (*SD*). *CI* = confidence interval; *LL* = lower level; *UL* = upper level; BDI-II = Beck Depression Inventory-II; RT = median reaction times; PE = percentages of error.

^a^Group x time interaction. ^b^refers to *n* = 20. ^c^refers to *n* = 31. ^d^refers to *n* = 15. ^e^refers to *n* = 32.

^I^MD+T+ = MD+T- > MD-T- (*p* < .001).

Regression analyses on the predictive value of lifetime trauma history (yes/no) for therapy outcome (*BDI-II* changes of depressive symptom severity and dropout status, respectively) yielded no associations (all *p*s ≥ .391). When, in a second step, adding respective facets of baseline cognitive functioning, more pronounced reductions of depressive symptom severity (*BDI-II*) emerged with smaller forward digit span, *b* = 1.48, 95% CI [0.07; 2.90], *p* = .041), while for all other measures of cognitive functioning, no predictive value emerged (all *p*s ≥ .059). Adding, in a third step, interaction terms of lifetime trauma history (yes/no) and baseline cognitive functioning did not predict CBT outcome regarding *BDI-II* and dropout status (all *p*s ≥ .058). Notably, robust regressions led to similar results.

## Discussion

The aim of the study was to assess associations of (i) lifetime trauma history according to the *DSM-IV* (American Psychiatric Association, 2007) and (ii) facets of cognitive functioning (i.e., WM, interference susceptibility, conflict adaptation, and OGM) as well as (iii) their interaction with CBT outcome among patients with MD. At baseline, more pronounced conflict adaptation emerged in individuals with MD and lifetime trauma history in contrast to non-exposed healthy controls, while autobiographical memory was found to be primarily affected in MD without lifetime trauma history compared to both control groups. Notably, individuals with MD with and without lifetime trauma history did not differ regarding treatment outcome, and the cognitive parameters proved stable over CBT. Exploratory analyses suggested no direct or interacting association of lifetime trauma history, and only a tentative one of forward digit span, but no other aspects of cognitive functioning with treatment outcome.

### Baseline Cognitive Functioning

On a cross-sectional level, the results support the role of lifetime trauma history for cognitive functioning in MD. While no differences emerged for interference susceptibility and WM, MD+T+ patients showed higher conflict adaptation of median RTs than MD-T- participants, with MD+T- and MD-T+ in between. This corresponds with previous findings from our group of more pronounced conflict adaptation in traumatized individuals with and possibly also without PTSD (Schindler et al., 2020; Steudte-Schmiedgen et al., 2014). However, as there also are suggested associations of conflict adaptation and depressive symptom severity (van Steenbergen et al., 2012), albeit without considering trauma history, further studies are desirable.

Interestingly, autobiographical memory yielded contrasting findings: MD+T- patients showed more pronounced OGM compared to the healthy control groups, corresponding with our previous findings of OGM in PTSD, but not trauma exposure *per se* (Schindler et al., 2020), and suggestions from reviews and meta-analyses (Moore & Zoellner, 2007; Ono et al., 2016; Sumner et al., 2010; Williams et al., 2007). Further, it supports the findings of Kuyken, Howell, and Dalgleish (2006) of OGM only in MD without (childhood) trauma history, but not those of Aglan et al. (2010) of OGM in MD with history of CSA. In sum, neither the results for conflict adaptation, nor those for OGM speak for a mere additive effect of trauma and MD on cognitive functioning, but rather for complex patterns with different impacts on different processes, and, potentially, different implications for clinical practice.

### Clinical and Cognitive Treatment Outcome Under Consideration of Lifetime Trauma History

In contrast to several previous studies particularly on childhood trauma (reviewed in Nemeroff, 2016; Teicher & Samson, 2013), our data suggest CBT to be equally effective in individuals with MD with and without the history of at least one traumatic event according to the *DSM-IV*. Several aspects may contribute to this divergence. Firstly, it is plausible that lifetime trauma, as examined in this study, does exert different effects than childhood trauma. Importantly, in our study, MD+T+ and MD+T- groups reported equal *CTQ* childhood maltreatment severity, and it is conceivable that this may have contributed to lacking group differences with respect to CBT effectiveness. Notably, also with respect to the *THQ*, the MD+T+ and the MD+T- groups did only differ on a descriptive level. However, it is important to consider that this instrument refers to the number and frequency of *potentially* traumatic events, for which the presence of the complete *DSM-IV* criteria are not checked. In order to better understand the role of childhood and adulthood trauma for CBT effectiveness, studies explicitly contrasting individuals with MD (i) without lifetime trauma, (ii) with exclusively childhood, and (iii) with exclusively adulthood trauma as defined by the current diagnostic criteria are necessary. Furthermore, treatment differences might have played a role. Most prominently, the majority of studies reporting similar therapy outcome for MD with and without (particularly childhood) trauma history had applied combined psychotherapy and antidepressant medication (Lewis et al., 2010; Miniati et al., 2010; Nemeroff et al., 2003; but Asarnow et al., 2009), as was the case for approximately half of our sample. Further, we cannot rule out whether, in our study, trauma status had led to slight individual treatment adaptations by the responsible therapists. This might, for instance, have led to combined modifications of trauma-related and -unrelated automatic thought patterns, or the encouraging of restarting activities avoided after the trauma during behavioral interventions within the context of the utilized CBT manuals (Hautzinger, 1998, 2008). Thus, future studies applying more strictly manualized CBT and investigating larger MD groups with and without medication intake are required.

Additionally, the results corroborate previous findings of cognitive alterations in MD being highly stable over CBT (reviewed in Köhler et al., 2015; Moore & Zoellner, 2007; Snyder & Hankin, 2019), and of this to be irrespective of trauma history. While WM improved from baseline to follow-up, this is presumably attributable to practice/habituation effects, as it also concerned MD-T- individuals. As cognitive impairments are assumed to be associated with worse psychosocial functioning and increased relapse risk in MD (Rock et al., 2014), the continuous finding of this to not be adequately addressed by CBT shows the necessity to strive for “cognitive”, next to clinical remission in MD (Bernhardt et al., 2019; Bortolato et al., 2016). For example, this might be achieved by directly targeting cognitive functioning during MD-centered CBT. While research on EF training in MD is still in its infancy (for a meta-analysis, see, e.g., Motter et al., 2016), there are promising results that OGM, as well as MD symptomatology itself may be influenced by interventions directly focusing on autobiographical recall, albeit with long-term stability still questionable (for a meta-analysis, see Barry, Sze, & Raes, 2019).

The exploratory predictive analyses on lifetime trauma history and cognitive functioning for CBT do not provide clear results from which robust next steps could be derived. What can be clearly stated as of now is that there, again, was no evidence for a relevant role of lifetime trauma history. Further, only a singular association with cognitive parameters emerged, suggesting smaller WM to be associated with more pronounced depressiveness-related CBT effects. In sum, this pattern, albeit stemming from a very small sample size, supports the findings of Goodkind et al. (2016) on interference susceptibility, but stands at variance with those of Sumner et al. (2010) suggesting a predictive role of autobiographical memory specificity in MD. Future studies are needed to follow up on autobiographical memory in this context, or investigate whether other cognitive markers might be more suitable to predict clinical outcome after standardized psychotherapeutic/pharmacological treatment (e.g., Groves et al., 2018) with or without taking trauma history into account.

### Strengths, Limitations, and Outlook

One central strength of the study is the naturalistic, highly ecologically valid study design. While the inclusion of a waiting control group of MD+T+/MD+T- patients not receiving CBT was impossible for ethical reasons, the fact that a healthy control group was studied longitudinally alongside the MD individuals is a further major strength, as it allowed the separation of CBT-associated and mere practice effects on cognitive functioning. However, limitations resulting from the naturalistic design are the heterogeneous manifestations of psychopathology and medication and the group differences in educational status and smoking. Further limitations include the lack of an objective, observer-rated outcome of depressiveness (e.g., the Hamilton Rating Scale for Depression; Hamilton, 1960), as well as the small sample sizes and the thus reduced statistical power for detecting especially interactive relationships. However, the fact that the vast majority of associations were confirmed in robust analyses corroborates the validity of the findings. Finally, behavioral tasks established in cognitive psychology, such as the ones used in our study, are characterized by task impurity, which describes the impossibility of assessing “pure” cognitive processes without simultaneously eliciting others (Miyake et al., 2000; e.g., Scott et al., 2015). In order to maximize transparency in data reporting, we chose to report subscale scores of the cognitive tasks for which different properties are discussed (Botvinick et al., 2004; Wechsler, 1997; Williams & Broadbent, 1986). In addition, we acknowledge that for any of the assessed tasks, additional cognitive processes such as processing speed, attention, and motivation – while not directly studied – are inevitably involved.

### Conclusions

In conclusion, the study is the first to examine lifetime trauma history, cognitive functioning, and their interaction in the context of CBT in patients with MD. On a cross-sectional level, conflict adaptation and autobiographical memory specificity emerged to be differentially affected in MD with and without lifetime trauma history. Contrary to previous research on childhood trauma, we found no evidence for a differential treatment response in patients with MD with and without lifetime trauma history as defined by the *DSM-IV*. Further, the cognitive parameters were stable over CBT, and only a singular predictive association of forward digit span, but no other facets of baseline cognitive functioning, lifetime trauma history, or their interaction with treatment outcome emerged. These insights into the interaction between lifetime trauma history and cognitive functioning provide unique extensions for research on MD psychopathology and treatment and underline the relevance of “cognitive” remission (Bernhardt et al., 2019; Bortolato et al., 2016). For achieving this aim, further research is required to allow more profound, neuroscience-informed diagnostic processes and personalized, multi-modal treatment approaches depending on patients’ individual manifestation of cognitive functioning (De Raedt, 2020).

## Supplementary Materials

The Supplementary Materials contain the following items (for access see Index of Supplementary Materials below):

- Supplement 1 (Lifetime trauma history and mental disorder comorbidities)
- Supplement 2 (Baseline demographic, health-related, and clinical characteristics of patients with major depression with (MD+T+) and without (MD+T-) as well as controls without (MD-T-) lifetime trauma history available for longitudinal analyses)

10.23668/psycharchives.5073 Supplement 1 Supplementary materials to "Lifetime trauma history and cognitive functioning in major depression and their role for cognitive-behavioral therapy outcome" [Additional information]

SchindlerL.
StalderT.
KirschbaumC.
PlessowF.
SchönfeldS.
HoyerJ.
TrautmannS.
WeidnerK.
Steudte-SchmiedgenS.
 (2021). Supplementary materials to "Lifetime trauma history and cognitive functioning in major depression and their role for cognitive-behavioral therapy outcome"
[Additional information]. PsychOpen. 10.23668/psycharchives.5073
PMC966723036398101
