# Supplementary material for: Lifetime Trauma History and Cognitive Functioning in Major Depression and Their Role for Cognitive-Behavioral Therapy Outcome
Source: Clin Psychol Eur. 2021 Sep 30;3(3):e4105. doi: 10.32872/cpe.4105 (PMC9667230; doi:10.32872/cpe.4105)
Supplement: Supplement 1 [file cpe-03-4105-s01.pdf]

Supplementary materials: Lifetime trauma history and cognitive functioning in major depression and their role for cognitive-behavioral therapy outcome

Lena Schindler[1], Tobias Stalder[2], Clemens Kirschbaum[1], Franziska Plessow[3], Sabine Schönfeld[1,4], Jürgen Hoyer[5], Sebastian Trautmann[5,6], Kerstin Weidner[7], Susann Steudte-Schmiedgen[1,7]

[1] Faculty of Psychology, Technische Universität Dresden, Germany
[2] Department Erziehungswissenschaften und Psychologie, Universität Siegen, Germany
[3] Neuroendocrine Unit, Department of Medicine, Massachusetts General Hospital and Harvard Medical School, Boston, MA, USA
[4] Department of Psychology, Lund University, Sweden
[5] Institute of Clinical Psychology and Psychotherapy, Technische Universität Dresden, Germany
[6] Department of Psychology, Medical School Hamburg, Germany
[7] Department of Psychotherapy and Psychosomatic Medicine, Medical Faculty Carl Gustav Carus, Technische Universität Dresden, Germany

Manuscript published in Clinical Psychology in Europe: https://doi.org/10.32872/cpe.4105

Supplement 1: Participants and procedures: Lifetime trauma history and mental disorder comorbidities

A heterogeneity of lifetime traumatic events was reported in the exposed groups, including accidents (*n* = 15), natural disasters (*n* = 7), physical assaults (*n* = 13), sexual assaults (*n* = 8), war/torture (*n* = 1), life-threatening illness (*n* = 14), and other traumatic events (*n* = 18) (e.g., death of a family member or close friend, *n* = 7). The majority of individuals (76.3%) reported that more than five years had passed since the traumatic event.

Supplement 2: Baseline demographic, health-related, and clinical characteristics of patients with major depression with (MD+T+) and without (MD+T-) as well as controls without (MD-T-) lifetime trauma history available for longitudinal analyses

Participants' characteristics	MD+T+ (*n* = 21)	MD+T- (*n* = 16)	MD-T- (*n* = 34)	Test Statistic	*p*
Demographics					
Age (*M*, *SD*)	35.52 (10.14)	40.75 (12.66)	34 (13.42)	$F_{2, 68} = 1.64$	.202
Female sex (%)	16 (76.2)	12 (75)	29 (85.3)	$X^2_2 = 1.04$	.593
Highest educational status				$X^2_6 = 9.65$	.14
Academic degree (%)	13 (18.6)	0 (0)[a]	9 (26.5)		
Professional training or college degree (%)	22 (31.4)	7 (46.7)[a]	10 (29.4)		
A level (%)	26 (37.1)	4 (26.7)[a]	13 (38.2)		
High school diploma or lower (%)	3 (14.3)	4 (26.7)[a]	2 (5.9)		
Smoking (%)	4 (19)	7 (43.8)	8 (23.5)	$X^2_2 = 3.18$	.204
Physical disease (%)	11 (52.4)	7 (43.8)	9 (26.5)	$X^2_2 = 3.99$	.136
Regular medication (%)	10 (47.6)	12 (75)	7 (20.6)	$X^2_2 = 13.9$	.001
Psychiatric (%)[b]	9 (42.9)	9 (56.3)	0		
Non-psychiatric (%)	4 (19)	7 (43.8)	7 (32)		
THQ number of A1 traumatic events (*M*, *SD*)	4.33 (2.56)	2.69 (1.74)	1.26 (1.21)	$F_{2, 68} = 18.61$	< .001[I]
THQ frequency of A1 traumatic events (*M*, *SD*)	7.2 (6.14)[c]	6.19 (5.47)	2.41 (3.4)	$F_{2, 67} = 7.33$	.001[II]
CTQ Score (*M*, *SD*)	37.62 (10.17)	38.56 (12.19)	29.09 (5.24)	$F_{2, 68} = 9.31$	< .001[III]

Abbreviations: *THQ = Trauma History Questionnaire*, *CTQ = Childhood Trauma Questionnaire*
[a] refers to *n* = 15, [b] included antidepressants (*n* = 18), neuroleptics (*n* = 2), anticonvulsives (*n* = 1), [c] refers to *n* = 20
[I] MD+T+ > MD+T- > MD-T- (*ps* ≤ .036), [II] MD+T+ = MD+T- > MD-T- (*ps* ≤ .035), [III] MD+T+ = MD+T- > MD-T- (*ps* ≤ .002)
